# Dual targeting of ANGPT1 and TGFBR2 genes by miR-204 controls angiogenesis in breast cancer

**DOI:** 10.1038/srep34504

**Published:** 2016-10-05

**Authors:** Ali Flores-Pérez, Laurence A. Marchat, Sergio Rodríguez-Cuevas, Verónica Bautista-Piña, Alfredo Hidalgo-Miranda, Elena Aréchaga Ocampo, Mónica Sierra Martínez, Carlos Palma-Flores, Miguel A. Fonseca-Sánchez, Horacio Astudillo-de la Vega, Erika Ruíz-García, Juan Antonio González-Barrios, Carlos Pérez-Plasencia, María L. Streber, César López-Camarillo

**Affiliations:** 1 Posgrado en Ciencias Genómicas, Universidad Autónoma de la Ciudad de México, Ciudad de México, México; 2 Programa en Biomedicina Molecular y Red de Biotecnología, Escuela Nacional de Medicina y Homeopatía, Instituto Politécnico Nacional, Ciudad de México, México; 3 Instituto de Enfermedades de la Mama, FUCAM, Ciudad de México, México; 4 Laboratorio de Genómica, Instituto Nacional de Medicina Genómica, Ciudad de México, México; 5 Departamento de Ciencias Naturales, Universidad Autónoma Metropolitana, Ciudad de México, México; 6 Laboratorio de Genética y Diagnóstico Molecular, Hospital Juárez, Ciudad de México, México; 7 Departamento de Genética Humana, Hospital General de Mexico “Dr Eduardo Liceaga”, Ciudad de México, México; 8 Laboratorio de Investigación en Cáncer Translacional y Terapia Celular, Centro Médico Siglo XXI, Ciudad de México, México; 9 Laboratorio de Medicina Translacional, Instituto Nacional de Cancerología, Ciudad de México, México; 10 Laboratorio de Medicina Genómica, Hospital Regional 1 de Octubre ISSSTE, Ciudad de México, México; 11 Laboratorio de Genómica, Instituto Nacional de Cancerología, Ciudad de México, México; Universidad Nacional Autónoma de México UNAM, FES-Iztacala, UBIMED, Tlalnepantla, Estado de México, México; 12 Laboratorio de Investigación Experimental y Animal. Instituto Nacional de Ciencias Médicas y Nutrición Salvador Zubirán, Ciudad de México, México

## Abstract

Deregulated expression of microRNAs has been associated with angiogenesis. Studying the miRNome of locally advanced breast tumors we unsuspectedly found a dramatically repression of miR-204, a small non-coding RNA with no previous involvement in tumor angiogenesis. Downregulation of miR-204 was confirmed in an independent cohort of patients and breast cancer cell lines. Gain-of-function analysis indicates that ectopic expression of miR-204 impairs cell proliferation, anchorage-independent growth, migration, invasion, and the formation of 3D capillary networks *in vitro*. Likewise, *in vivo* vascularization and angiogenesis were suppressed by miR-204 in a nu/nu mice model. Genome-wide profiling of MDA-MB-231 cells expressing miR-204 revealed changes in the expression of hundred cancer-related genes. Of these, we focused on the study of pro-angiogenic ANGPT1 and TGFβR2. Functional analysis using luciferase reporter and rescue assays confirmed that ANGPT1 and TGFβR2 are novel effectors downstream of miR-204. Accordingly, an inverse correlation between miR-204 and ANGPT1/TGFβR2 expression was found in breast tumors. Knockdown of TGFβR2, but not ANGPT1, impairs cell proliferation and migration whereas inhibition of both genes inhibits angiogenesis. Taken altogether, our findings reveal a novel role for miR-204/ANGPT1/TGFβR2 axis in tumor angiogenesis. We propose that therapeutic manipulation of miR-204 levels may represent a promising approach in breast cancer.

Over the last decade, microRNAs (miRNAs) have emerged as a prominent class of novel negative regulators of gene expression^1^. These non-coding single-stranded RNAs are evolutionary conserved, and function as guide molecules in posttranscriptional gene silencing by partially complementing with the 3′ untranslated region (UTR) of the target, resulting in mRNA degradation or translational repression^2^. Remarkably, aberrant expression of miRNAs may contribute to development of diverse neoplasia and in some cases correlates with clinical-pathological features of tumors representing potential prognostic markers and novel therapeutic targets in cancer^3^. One mechanism by which miRNAs may induce tumorigenesis is by affecting the production of angiogenic factors and neovascularization processes. Angiogenesis is a complex mechanism of neovascular formation from pre-existing blood vessels. Pathological formation of new blood vessels confers advantages to tumor growth and metastasis establishment; therefore molecular mechanisms of angiogenesis are major issues in the understanding of cancer progression, and constitute an important therapeutic target in cancer^4^. Recent investigations into molecular mechanisms of tumor angiogenesis have led to the identification of novel angiogenic targets, which have been translated into the development of promising anti-vascular agents^5^. In neoplasms, tumor-derived factors promote angiogenesis through the activation of multiple cellular processes such as cell division, growth, migration and invasion^6^. Key pro-angiogenic factors that enhance endothelial cell migration and capillary-like structure formation include the hypoxia-inducible factor 1-alpha (HIF1α), the vascular endothelial growth factor A (VEGFA), the transforming growth factor beta-1 (TGFβ1), the angiopoietins (ANGPT1 and ANGPT2), the plasminogen (PLG), and endostatins. Importantly, several genes involved in angiogenesis-dependent growth of tumors are regulated by miRNAs^7^,^8^,^9^,^10^. Therefore, deciphering the miRNAs network responsible for the modulation of angiogenesis might lead to discovery of novel therapeutic approaches for cancer. Nonetheless, the potential biological role of most miRNAs in angiogenesis regulation of breast cancer is poorly understood. Here, we analyzed the microRNome of primary breast tumors and found that miR-204 was suppressed in clinical specimens. Furthermore, we provide experimental data indicating that miR-204 inhibits diverse hallmarks of breast cancer, in particular angiogenesis through the targeting of key pro-angiogenic genes.

## Results

### Global miRNAs profiling of locally invasive breast tumors

**Figure 1:**
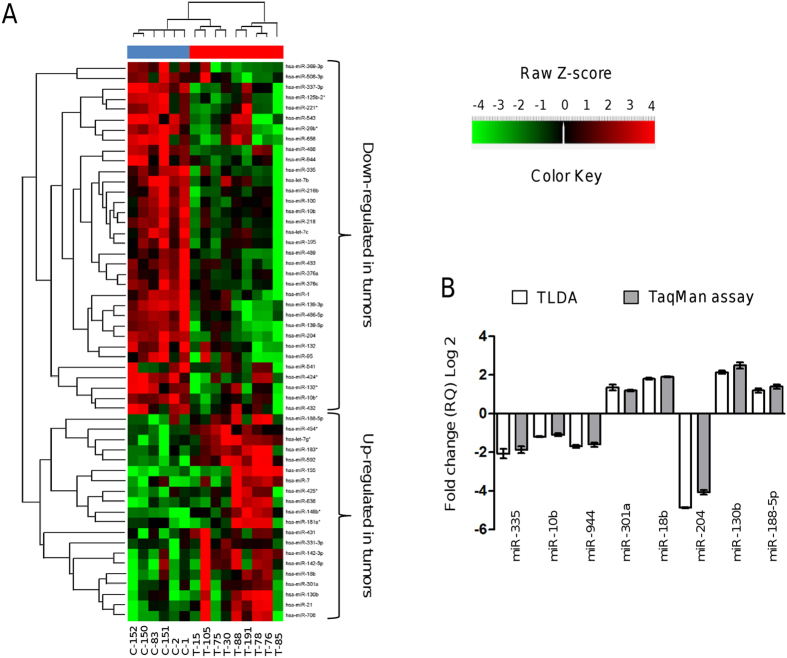
Expression profiling of microRNAs in locally invasive breast tumors and normal tissues. (**A**) Unsupervised hierarchical clustering analysis displaying the differential expression of microRNAs in breast tumors relative to normal tissues from TLDA. The heat map (Spearman correlation, Euclidean distance) represents a cluster analysis of the logarithm of transformed ΔΔCt values of the differentially expressed microRNAs. Color key: upregulated microRNAs (red); downregulated microRNAs (green). Blue upper bar, normal tissues; red upper bar, tumor tissues. (**B**) Validation of expression levels of eight microRNAs using Taqman qRT-PCR assay (grey) in comparison with data obtained from TLDA (white). Data were expressed as mean ± S.D.

In order to identify miRNAs differentially expressed between primary breast tumors and normal mammary tissues, we profiled 667 mature miRNAs using stem-loop reverse transcription-quantitative PCR (RT-qPCR) in TaqMan low-density arrays (TLDAs). Tumors were collected from nine patients with locally invasive ductal breast carcinomas (discovery cohort). An overview of the clinical and pathological features of tumors and patients included in this study is given in Table 1. After comparative 2-ΔΔCt analyses, we identified a total of 54 miRNAs significantly deregulated in clinical specimens (|log_2_(T/N)| > 1.0; *p* < 0.05). Of these, 34 miRNAs were downregulated and 20 were upregulated (Table 2). This panel of miRNAs was able to separate the tumors group from the normal samples in the 2-way unsupervised hierarchical cluster shown in Fig. 1A indicating that differences among samples were not due to heterogeneity or the presence of different cell types in biopsies. A group of 23 miRNAs (four upregulated and 19 downregulated) were homogeneously expressed across the set of tumors (see Supplementary Table 1). These include miRNAs with known roles in breast cancer development such as miR-7, miR-10b, miR-21, miR-100, miR-155, miR-195, miR-221, and miR-218. In addition, we found that a large number of predicted target genes belong to key cellular pathways that might modulate the hallmarks of cancer, including MAP kinases, focal adhesion, WNT, TGF-β, and ERBB signaling (see Supplementary Table 2). A number of 14 miRNAs were located in chromosomal regions frequently deleted or amplified in breast cancer. In order to corroborate the differential expression of miRNAs identified by TLDAs, eight deregulated miRNAs were analyzed by RT-qPCR in biological replicates. Results showed that expression levels of miR-335, miR-10b, miR-944, miR-301a, miR-18b, miR-204, miR-130b, and miR-188-5p were similar in both assays (Fig. 1B). To further validate the arrays results, we analyzed a larger and independent dataset of miRNAs expression in 776 breast tumors and matched adjacent tissues (validation cohort) obtained from The Cancer Genome Atlas (TCGA). Results showed that of the 54 miRNAs that we previously found with differential expression in the discovery cohort 33 were reported in TCGA sets. Of these, 29 miRNAs exhibits expression levels very similar to those found in our miRNome analysis, whereas only four miRNAs showed discordant expression between the discovery and validation cohort (*p* < 0.0001; see Supplementary Fig. 1).

### MicroRNA-204 is suppressed in breast tumors and cancer cell lines

**Figure 2:**
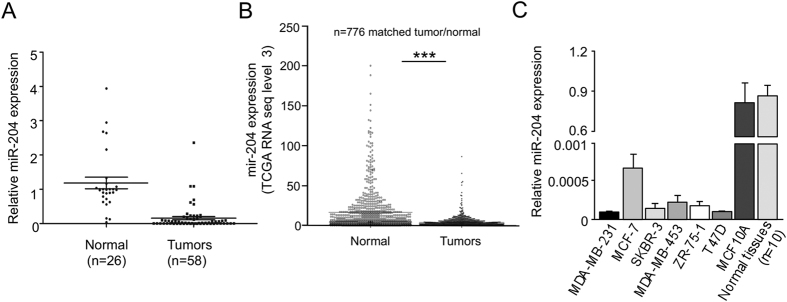
MiR-204 is suppressed in breast tumors and cancer cell lines. (**A**) qRT-PCR assays for miR-204 relative expression in non-tumoral adjacent breast tissues and breast tumors. (**B**) MiR-204 expression in 776 breast tumors and matched adjacent normal samples from TCGA datasets (***p < 0.0001). (**C**) qRT-PCR assays for miR-204 expression in breast cancer cell lines. Non-tumorigenic MCF-10A cell line and normal adjacent tissues pool (n = 10) were used as controls. Data were normalized with the endogenous small-nucleolar RNU44. Bars represent the mean of three independent experiments ± S.D.

The miRNAs profiling of locally invasive breast tumors allowed us to evidence that, in particular, miR-204 was suppressed. To study the biological relevance of miR-204, we measured its expression by RT-qPCR in clinical specimens obtained from an independent cohort of 58 breast cancer patients. Clinical features of breast tumors including hormonal receptors status, tumor size, histology, clinical stage, and tumor grade are summarized in Table 3. Results indicated that miR-204 expression was significantly low (*p* < 0.05) in breast tumors in comparison with normal adjacent tissues (Fig. 2A). No association between miR-204 levels and the status of estrogen, progesterone and HER2/neu receptors was found. We further performed a validation analysis using 776 breast tumors and matched normal adjacent samples from the TGCA datasets. Results confirmed that miR-204 was significantly (*p* < 0.0001) suppressed in breast tumors in comparison to normal tissues in this large cohort of patients (Fig. 2B). Moreover, we found that miR-204 was severely downregulated in MCF-7, MDA-MB-231, MDA-MB-45, ZR-45, and T47-D breast cancer cell lines when compared with non-tumorigenic MCF-10A cells and normal tissues (Fig. 2C).

### MicroRNA-204 inhibits cell proliferation and anchorage-independent growth

**Figure 3:**
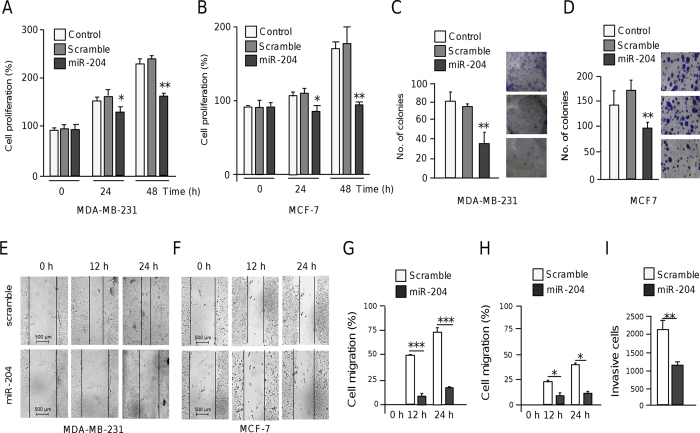
MiR-204 represses cell proliferation, growth and migration. (**A,B**) MTT assays of MDA-MB-231 (**A**) and MCF-7 cells (**B**) transfected with pre-miR-204 (30 nM) or pre-miR-negative control (scramble) at 48 h and 24 h post-transfection. (**C,D**) Graphical representation of colony-forming assays for MDA-MB-231 (**C**) and MCF-7 cells (**D**) transfected with pre-miR-204 or scramble. (**E**,**F**) Scratch/wound-healing assays of MDA-MB-231 (**E**) and MCF-7 (**F**) cell monolayers treated with pre-miR-204 (30 nM), or scramble for 12 h and 24 h. (**G,H**) Transwell cell migrating assays of MDA-MB-231 (**G**) and MCF-7 (**H**) cells treated as above. (**I**) Matrigel invasion assays of MDA-MB-231 cells transfected with pre-miR-204 (30 nM). Results shown are the mean of three independent experiments +/− SD. *p < 0.05, **p < 0.01 and ***p < 0.001 compared to controls. Bars represent the mean of three independent experiments ± S.D.

We next wondered whether ectopic expression of miR-204 could have tumor suppressor effects *in vitro*. Results of MTT assays showed that the growth of both MDA-MB-231 and MCF-7 cells transfected with miR-204 was significantly (*p* < 0.05) decreased in comparison with non-transfected and scramble transfected control cells (Fig. 3A,B). Moreover, colony formation assays indicated that anchorage-independent growth was attenuated (*p* < 0.05) by miR-204 in both cell lines relative to controls (Fig. 3C,D).

### MicroRNA-204 impairs cell migration and invasion

To evaluate the contribution of miR-204 in cell migration and invasion we restored its expression in triple negative MDA-MB-231 (highly metastatic) and estrogen responsive MCF-7 (poorly invasive) breast cancer cell lines and then performed scratch/wound-healing and transwell assays. Results showed that monolayer restoration at 24 h was significantly (*p* < 0.05) delayed by 82% and 72% in MDA-MB-231 and MCF-7 cells respectively in comparison with non-transfected control cells (Fig. 3E,F). Furthermore, we found that the number of migratory cells at 24 h was reduced (*p* < 0.05) in miR-204-expressing MDA-MB-231 cells in comparison with control (Fig. 3G,H). Congruently, restoration of miR-204 levels also attenuated the ability of MDA-MB-231 cells to invade matrigel chambers (Fig. 3I).

### MicroRNA-204 impairs angiogenesis *in vitro*

**Figure 4:**
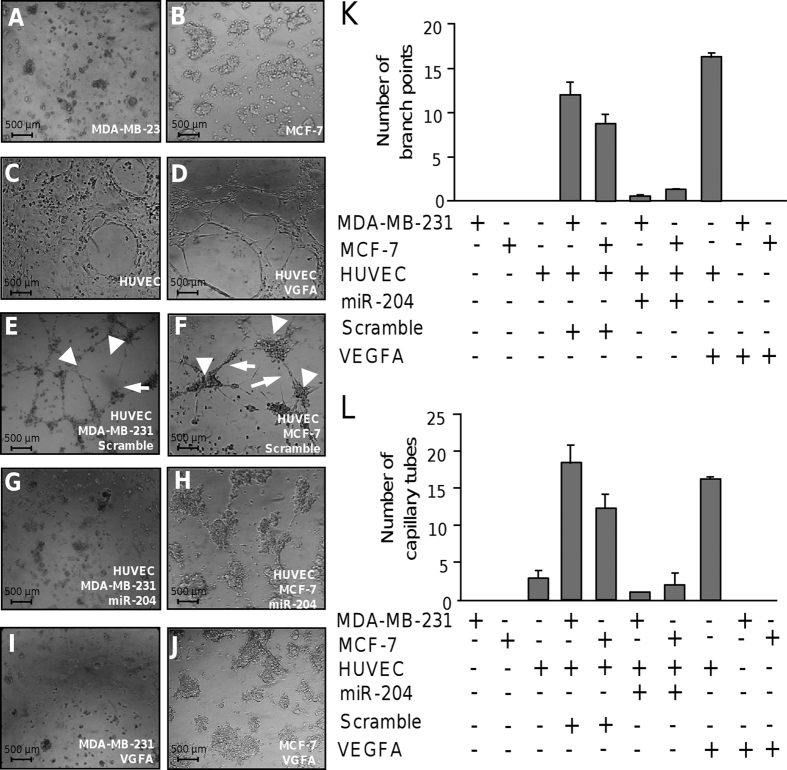
MiR-204 inhibits angiogenesis. Capillary tubes development was evaluated *in vitro* using co-cultures of HUVEC and breast cancer cells. (**A**) MDA-MB-231 and (**B**) MCF-7 cells plated onto matrigel with serum free medium. (**C**) HUVEC plated into matrigel with endothelial complete medium. (**D**) Monoculture of HUVEC in presence of VEGFA (10 ng/ml). (**E**) Co-culture of HUVEC with MDA-MB-231 or (**D**) MCF-7 cells transfected with scramble and plated into matrigel with serum free medium for 24 h. (**G**) Co-culture of HUVEC with MDA-MB-231 or (**H**) MCF-7 cells transfected with miR-204 (30 nM) and plated into matrigel with serum free medium for 24 h. (**I**) Mono-cultures of MDA-MB-231 or (**J**) MCF-7 cells in presence of VEGFA (10 ng/ml). Graphical representation of quantification of branch points (**K**) and capillary tubes (**L**) number after 24 h of co-cultures. Data were obtained by two different observers. Results shown are the mean of three independent experiments ± S.D. Arrowheads indicate branch points. Arrows denote capillary-like tubes structures.

Tumor progression requires a sustained angiogenesis. As the role of miR-204 in angiogenesis is largely unknown in breast cancer, we investigated its contribution in this cancer hallmark. We carried out tube formation assays using human umbilical vein endothelial cells (HUVEC), which is one of the simple but well-established *in vitro* angiogenesis assays based on the ability of endothelial cells to form three-dimensional (3D) capillary-like tubular structures. Co-cultures of HUVEC with MDA-MB-231 or MCF-7 breast cancer cells in different conditions were performed. As expected MDA-MB-231, MCF-7 and HUVEC cells alone did not form tubules-like structures (Fig. 4A–C). In contrast, a strong angiogenic effect was observed in HUVEC cells treated with recombinant VEGFA used as positive control (Fig. 4D). Typical HUVEC tubular networks on the matrigel were observed at 24 h. Co-incubation of HUVEC with MDA-MB-231 or MCF-7 cells transfected with scramble control also resulted in an angiogenic behavior as the number of endothelial cells branch points and capillary tubes were increased in comparison with monoculture controls (Fig. 4E,F,K,L). Interestingly, co-culture of HUVEC with MDA-MB-231 or MCF-7 cells transfected with miR-204 resulted in a marked inhibition of endothelial cell tubules and branch point formation, and the tubular networks were disrupted at 24 h (Fig. 4G,H,K,L). Taken altogether these data indicate that miR-204 inhibits the angiogenesis-induced by breast cancer cells in endothelial cells.

### MicroRNA-204 inhibits vascularization and angiogenesis *in vivo*

**Figure 5:**
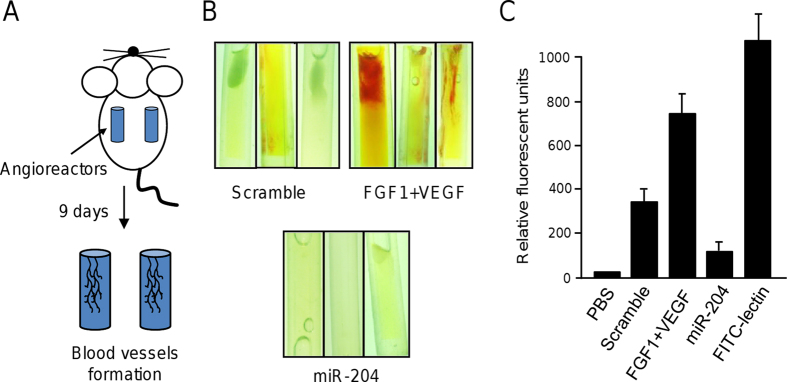
MiR-204 inhibits blood vessels formation *in vivo.* (**A**) Directed *in vivo* angiogenesis assay. (**B**) Representative images of angioreactors extirpated from nude mice 9 days after implantation. Inductor of angiogenesis VEGF and FGF1 or MDA-MB-231 cells transfected with miR-204 or scramble control were placed into angioreactors as described in Methods section. (**C**) Fluorometric quantification of blood vessels formation using FITC-lectin detection assay. Experiments were performed by duplicate tree times.

Then we asked if miR-204 have an effect in blood vessels formation in a nude mice model. A direct *in vivo* angiogenesis assay (DIVAA) was setup as describes in Methods. In this assay, the cellular *in vivo* vascularization was evaluated using transplanted angioreactors, which provides a system to determine an effective response to angiogenic modulating factors. Angioreactors containing basement membrane extract (BME) mixed with VEGF/FGF1 (positive control), scramble transfected cells (negative control), or miR-204 transfected cells (test) were implanted in nude mice. After nine days of implantation angioreactors were removed and vascularization analyzed (Fig. 5A). Data showed a significant blood vessels infiltration in angioreactors containing pro-angiogenic factors VEGF and FGF1. In contrast, angioreactors mixed with MDA-MB-231 cells transfected with scramble showed a significant very low infiltration (Fig. 5B upper panel). Remarkably, blood vessels infiltration was severely impaired in angioreactors containing miR-204 transfected breast cancer cells (Fig. 5B bottom panel) in comparison to controls, indicating that miR-204 significantly inhibits angiogenesis *in vivo*.

### MicroRNA-204 modulates genes involved in cell proliferation, migration and angiogenesis

**Figure 6:**
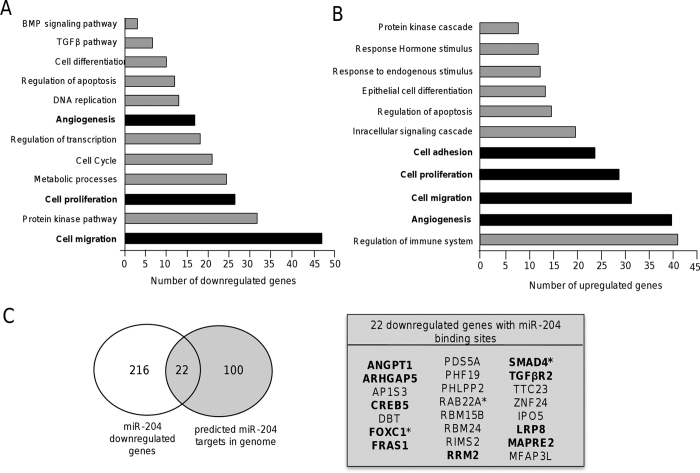
Classification of genes modulated by miR-204. The pathways and biological processes analysis using gene ontology terms is shown. (**A**) Downregulated and, (**B**) upregulated genes. Cellular processes of particular interest in the present study are denoted in bold. (**C**) Venn diagram comparing the number of genes modulated by miR-204 overexpression (white circle) and predicted as miR-204 targets (grey circle) by TargetScan and Pic-tar programs. Circles intersection indicates the 22 genes identified in both analyses. Genes related to cell proliferation, migration and angiogenesis are in bold. Previously validated targets of miR-204 are indicated with asterisks.

In order to identify potential target genes of miR-204 associated to the inhibition of the cancer hallmarks described above, we carried out a transcriptional profiling of MDA-MB-231 cells that ectopically express miR-204 (see Supplementary Fig. 2) using DNA microarrays. Data from two biological replicates were analyzed, normalized, and raw *p*-values adjusted. Only the genes with a significant fold change (FC > 1.5; *p* < 0.05) were included in this analysis. Genome-wide analysis showed that 549 genes (311 upregulated and 238 downregulated) were significantly modulated (see Supplementary Table 3). To validate DNA microarrays data, the mRNA expression of ten selected genes was analyzed by RT-qPCR using specific oligonucleotides (see Supplementary Table 4). Triplicates were performed for each sample and for each gene. In all cases, the mRNA expression values obtained by RT-qPCR were similar to those found by DNA microarrays analysis (see Supplementary Fig. 3). Classification of genes based on Gene ontology categories indicated that a number of genes were involved in cellular processes and pathways frequently deregulated in human cancers (Fig. 6A,B). Of the 238 downregulated genes a subset of 22 genes contains potential miR-204 binding sites at their 3′UTR as predicted by TargetScan and Pic-Tar programs (Fig. 6C), and three genes (FOXC1, RAB22A, SMAD4) were previously reported as truly miR-204 targets. Interestingly, these repressed genes are involved in functions related to cell proliferation, migration, apoptosis and angiogenesis (see Supplementary Table 5). In agreement with the suppressive role of miR-204 in cell proliferation and angiogenesis, the pro-angiogenic angiopoietin-1 (ANGPT1) and the transforming growth factor β receptor type 2 (TGFβR2) genes were downregulated in MDA-MB-231 cells with restored expression of miR-204. In particular, these genes play key roles in cell migration, invasion and in the activation of angiogenesis program of tumor cells.

### MicroRNA-204 targets the angiogenesis-related ANGPT1 and TGFβR2 genes

**Figure 7:**
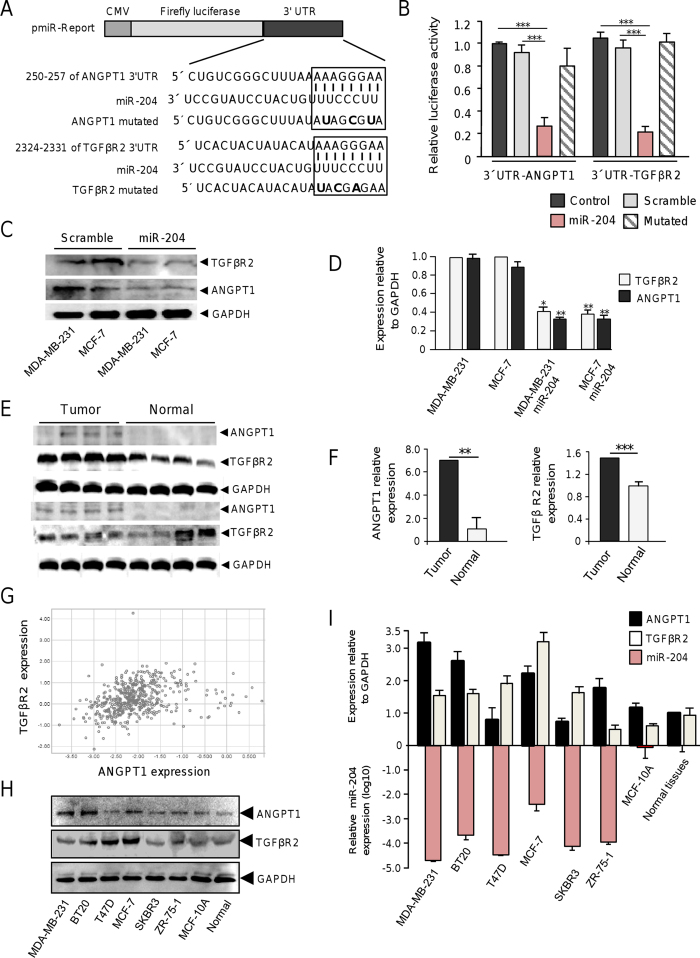
MiR-204 targets ANGPT1 and TGFβR2. (**A**) Schematic representation of p-miR report constructs containing the 3′UTR of ANGPT1 and TGFβR2 genes cloned downstream of luciferase gene. Seed sequences are indicated in boxes. Point mutations in the miR-204 binding sites of 3′UTR of ANGPT1 or TGFβR2 genes are denoted in bold. (**B**) Luciferase assays in MDA-MB-231 cells transfected with miR-204 and the constructs described in A. Cells transfected with p-miR report plasmid alone or not transfected were used as controls. Data represent the mean ± S.D. of three independent experiments. (**C**) Western blot of MDA-MB-231 and MCF-7 cells, non-transfected and transfected with pre-miR-204, using ANGPT1 (1:1000) and TGFβR2 (1:1000) primary antibodies. GAPDH antibodies were used as control. (**D**) Densitometric analysis of bands in panel C. Data were normalized using GAPDH expression. Images are representative of three independent experiments. (**E**) Western blots of two representative sets of breast tumors and non-tumoral tissues using ANGPT1 (1:1000) and TGFβR2 (1:1000) antibodies. GAPDH antibodies were used as control. Data were normalized using GAPDH expression. (**F**) Densitometric analysis of immunodetected bands in panel E. (**G**) Comparison of ANGPT1 and TGFβR2 expression in breast cancer using dataset from TCGA. (**H**) Western blot assays for ANGPT1 and TGFβR2 in breast cancer cell lines and normal tissues. (**I**) Densitometric analysis of immunodetected bands in **H** (upper bars), and miR-204 expression levels in the same breast cancer cell lines (bottom bars). Protein expression data were normalized using GAPDH. Data for miR-204 expression were normalized using RNU44. Bars are representative of three independent experiments ± S.D.

Increased expression of ANGPT1 and TGFβR2 promotes cell proliferation and angiogenesis in diverse types of tumors^11^,^12^, thus its reliable to propose that miR-204 may negatively regulate these cellular processes through direct targeting of both genes. To corroborate whether miR-204 can exerts posttranscriptional repression of ANGPT1 and TGFβR2, we used luciferase reporter gene assays. Nucleotides sequences corresponding to 3′UTR of either ANGPT1 or TGFβR2 genes were cloned downstream of the luciferase coding region of pmiR report vector (Fig. 6A). In addition, three point mutations in the predicted miR-204 binding site of the 3′UTR of ANGPT1 or TGFβR2 genes were included in the analysis. Recombinant pmiR-LUC-ANGPT1 and pmiR-LUC-TGFβR2 plasmids were transfected into MDA-MB-231 cells and luciferase activity was analyzed after 24 h. Data showed that ectopic expression of miR-204 and co-transfection of either pmiR-LUC-ANGPT1 or pmiR-LUC-TGFβR2 constructs resulted in a significantly reduction of the relative luciferase activity in comparison with controls (Fig. 7B). When mutated sequences of the 3′UTR of ANGPT1 and TGFβR2 genes were assayed, no significant changes in luciferase activity were found indicating that miR-204 binding was specific. Furthermore, Western blot assays corroborated that miR-204 restoration resulted in a significant decrease of the endogenous ANGPT1 and TGFβR2 proteins in both MDA-MB-231 and MCF-7 cells (Fig. 6C,D). In order to extend our observations, then we evaluated if miR-204 suppression in clinical breast tumors correlates with the increased expression of ANGPT1 and TGFβR2 proteins. Our results indicated that the mean expression of ANGPT1and TGFβR2 was increased (60%) in miR-204-deficient breast tumors in comparison with normal tissues (Fig. 6E,F). To further corroborate these findings, an integration-based approach was applied to compare the ANGPT1 and TGFβR2 expression in a larger cohort of breast cancer patients (n = 522) using datasets from TCGA. Results indicate that both ANGPT1 and TGFβR2 were downregulated in the half of samples (Fig. 6G). Furthermore, the expression of ANGPT1 and TGFβR2 was evaluated by Western blot in the six breast cancer cell lines which exhibits reduced miR-204 levels. We found higher ANGPT1 expression in MDA-MB-231, BT20, MCF-7, and ZR-75-1 cell lines, but not inT47D and SKBR3 cells, in comparison to non-tumorigenic MCF-10A and normal adjacent tissues (Fig. 6H). In a similar way high expression levels of TGFβR2 were observed in five breast cancer cell lines, except for ZR-75-1 cells, relative to MCF-10A and normal tissues. These data suggested the existence of an inverse correlation between the expression of mR-204 and ANGPT1 and TGFβR2 proteins in the majority of breast cancer cell lines tested.

### TGFβR2 gene silencing, but not ANGPT1, impairs cell proliferation and migration

**Figure 8:**
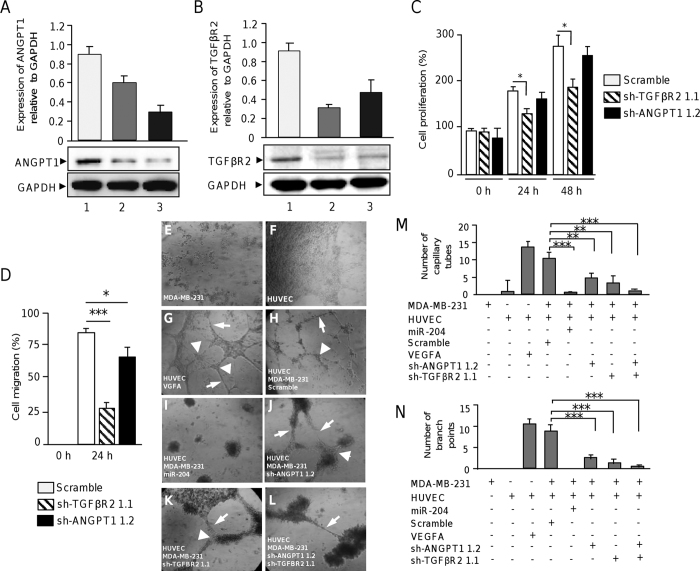
Effects of ANGPT1 and TGFβR2 targeted silencing in cell proliferation, migration and angiogenesis. (**A**) Western blot analysis for ANGPT1 expression in MDA-MB-231 cells transfected with scramble (lane 1), sh-ANGPT1 1.1 (lane 2) or sh-ANGPT1 1.2 (lane 3) constructs using ANGPT1 (1:1000) antibodies. Upper panel, densitometric analysis of immunodetected bands. (**B**) Western blot analysis for TGFβR2 in MDA-MB-231 cells transfected with scramble (lane 1), sh-TGFβR2 1.1 (lane 2) or sh-TGFβR2 1.2 (lane 3) constructs using TGFβR2 (1:1000) antibodies. Upper panel, densitometric analysis of immunodetected bands. Data were normalized using GAPDH expression. Images are representative of three independent experiments. (**C**) MTT assays and (**D**) Scratch/wound-healing assays of MDA-MB-231 cells transfected with scramble, sh-ANGPT1 1.1 or sh-TGFβR2 1.2 plasmids. (**E–N**) Capillary tubes development using co-cultures of HUVEC and breast cancer cells. (**E**) MDA-MB-231 and (**F**) HUVEC cells plated into matrigel with endothelial complete medium. (**G**) Monoculture of HUVEC in presence of VEGFA (10 ng/ml). (H) Co-culture of HUVEC with MDA-MB-231 transfected with scramble or with (**I**) miR-204 (30 nM). (**J**) Co-culture of HUVEC with MDA-MB-231 transfected cells with sh-ANGPT1 1.2, (**K**) sh-TGFβR2 1.1 or (**L**) both sh-ANGPT1 1.2 and sh-TGFβR2 1.1 plasmids. Graphical representation of (**M**) branch points, and (**N**) capillary tubes number after 24 h of co-cultures. Arrowheads indicate branch points. Arrows denote capillary-like tubes structures. Results shown are the mean of three independent experiments +/− SD. *p < 0.05, **p < 0.01 and ***p < 0.001 compared to controls. Bars represent the mean of three independent experiments ± S.D.

As we previously evidenced that miR-204 directly binds to 3′UTR of the pro-angiogenic ANGPT1 and TGFβR2 genes and inhibits its expression, we sought to determine if targeted inhibition of ANGPT1 and TGFβR2 could affect cell proliferation and migration. Therefore, we proceeded to knock-down its expression by RNA interference using two specific shRNAs targeting both the ANGPT1 and TGFβR2 genes (see Supplementary Table 6). The designed shRNAs dubbed as sh-ANGPT1 1.1, sh-ANGPT1 1.2, sh-TGFβR2 1.1 and sh-TGFβR2 1.2 were cloned in pSilencer vector. The constructs were individually introduced into MDA-MB-231 cells and protein expression was analyzed by Western blot 48 h after transfection. Results showed that the four short-hairpin sequences effectively down-regulate both the ANGPT1 and TGFβR2 expression (Fig. 8A,B). Densitometric analysis of immunodetected bands showed that gene silencing induced by sh-ANGPT1 1.2 and sh-TGFβR2 1.1 sequences was more effective since they suppressed ANGPT1 and TGFβR2 expression by 58% and 62%, respectively, thus they were selected for further experiments. GADPH used as a control, did not showed significantly expression changes between treatments. The effects of ANGPT1 and TGFβR2 silencing in cell proliferation were evaluated in MDA-MB-231 cells. Results of MTT assays showed that cell proliferation was significantly (*p* < 0.05) decreased in TGFβR2 -deficient cells at 24 h and 48 h in comparison with control (Fig. 8C). In contrast, we did not observed significant differences in cell proliferation in ANGPT1-deficient cells (Fig. 8C). Then, we sought to evaluate the effects of ANGPT1 and TGFβR2 knock-down in cell migration using scratch/wound healing assays. Our data indicate that cell monolayers restoration was significantly delayed at 24 h in both ANGPT1 (*p* < 0.05) and TGFβR2 (*p* < 0.001)-deficient cells relative to control (Fig. 8D) being more evident the inhibitory effect in the TGFβR2 silenced cells.

### Knockdown of ANGPT1 and TGFβR2 suppresses angiogenesis

In order to define if knockdown of ANGPT1 and TGFβR2 impairs angiogenesis we carried out tube formation assays as described above. As previously observed, co-incubation of HUVEC with MDA-MB-231 cells transfected with miR-204 resulted in a dramatical reduction of the number of endothelial cells branch points and capillary tubes in comparison with controls (Fig. 8E–G). Interestingly, co-culture of HUVEC with ANGPT1-deficient cells alters the typical morphology and development of endothelial cell tubules. Moreover, the number of capillary-like structures decreased up to 70%, whereas the number of branch points diminished up to 80% in comparison to HUVEC cells treated with VGFA used as positive control (Fig. 8F,G). Moreover, typical tubular networks on the matrigel were disrupted at 24 h. The inhibition of TGFβR2 produced a similar effect in angiogenesis, as we found that the formation of tubules was also compromised (Fig. 8E–G). An additive effect was observed when ANGPT1 and TGFβR2 genes were knock-down, as the number of capillary-like structures and the branch points diminished up to 95% in comparison to control. These changes were not due to alterations in cell viability of MDA-MB-231 with reduced expression of ANGPT1 and TGFβR2.

### Rescue of ANGPT1 and TGFβR2 in miR-204 expressing cells partially restore angiogenesis

**Figure 9:**
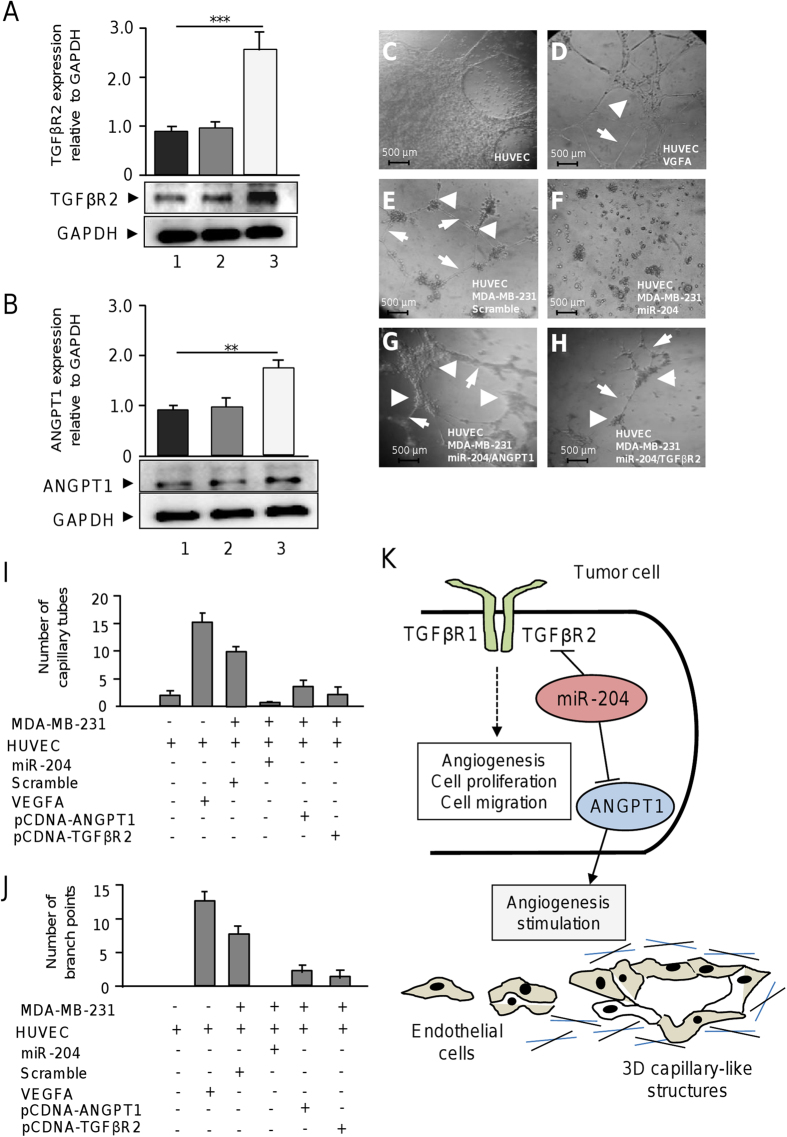
Rescue of ANGPT1 and TGFβR2 in miR-204 expressing cells partially restore angiogenesis. (**A**) Western blot analysis for TGFβR2 expression in (1) MDA-MB-231 cells treated with transfection agent, (2) cells transfected with empty vector and, (3) cells transfected with pCDNA3-TGFβR2 using anti-TGFβR2 (1:1000) antibodies. Upper panel, densitometric analysis of immunodetected bands. (**B**) Western blot analysis for ANGPT1 in (1) MDA-MB-231 cells treated with transfection agent, (2) cells transfected with empty vector and, (3) cells transfected with pCDNA3-ANGPT1 using anti-ANGPT1 (1:1000) antibodies. Upper panel, densitometric analysis of immunodetected bands. Data were normalized using GAPDH expression. (**C**) monoculture of HUVEC cells. (**D**) HUVEC cells treated with VEGFA (10 ng/ml). (**E**) co-culture of HUVEC and MDA-MB-231 cells transfected with scramble. (**F**) co-culture of HUVEC and MDA-MB-231 cells transfected with miR-204 precursor. (**G**) co-culture of HUVEC with MDA-MB-231 cells co-transfected with miR-204 and pcDNA3-ANGPT1 plasmid. (**H**) co-culture of HUVEC with MDA-MB-231 cells co-transfected with miR-204 and pcDNA3-TGFβR2 construct. Arrowheads indicate branch points. Arrows denote capillary-like tubes structures. (**I**) Graphical representation of the quantification of capillary tubes, and (**J**) branch points number after 24 h of co-cultures. Data were obtained by two different observers. Results shown are the mean of three independent experiments. Bars represent the mean of three independent experiments ± S.D. **p < 0.01, ***p < 0.001. (**C–H**) 3D capillary-like formation evaluated *in vitro* using co-cultures of HUVEC and breast cancer cells. (**K**) Working model of the miR-204 functions in angiogenesis of endothelial cells through the simultaneous targeting of ANGPT1 and TGFβR2 genes in breast cancer cells.

To obtain additional insights confirming the role of miR-204/ANGPT1/TGFβR2 axis in angiogenesis we performed rescue assays. The complete open reading frame of ANGPT1 and TGFβR2 genes were amplified and cloned into the pcDNA3 expression vector to overexpress the proteins in breast cancer cells. MDA-MB-231 cells were cotransfected with miR-204 and then with pcDNA3-ANGPT1 or pcDNA3-TGFβR2 constructs and *in vitro* angiogenesis assays were performed. Western blot assays confirmed that transfection of pcDNA3-ANGPT1 or pcDNA3-TGFβR2 result in significant increased levels of ANGPT1 and TGFβR2 in MDA-MB-231 cells expressing miR-204 (Fig. 9A,B). After that tube formation assays were performed as described above. As previously observed HUVEC control cells in the presence of VGFA form tubules-like structures which were abolished after 24 h coculture with MDA-MB-231 cells expressing miR-204 (Fig. 9D,F). Interestingly, co-incubation of HUVEC with MDA-MB-231 cells transfected with miR-204 and then rescued with pcDNA3-ANGPT1 resulted in an moderate increase of angiogenic behavior as the number of endothelial cells branch points and capillary tubes were significantly increased in comparison with controls represented by cells transfected with miR-204 alone (Fig. 9G,I,J). Likewise, transfection of pcDNA3-TGFβR2 in MDA-MB-231 cells expressing miR-204 resulted in an moderate increase in the number of endothelial cells branch points and capillary tubes in comparison with control (Fig. 9H,I, J). Taken altogether these data indicate that ANGPT1 and TGFβR2 are effectors of miR-204 and that its forced expression partially restores angiogenesis in breast cancer cells.

## Discussion

In this study, we analyzed the miRNome of a set of ductal breast tumors, and identified a signature of 54 miRNAs differentially expressed between tumors and normal tissues. Importantly, we validated the expression of 29 miRNAs in 776 breast tumors and matched adjacent tissues obtained from TCGA. In particular, we found that miR-204, a miRNA that is frequently repressed in human malignancies, was consistently downregulated in all the clinical specimens analyzed here. Importantly, other downregulated miRNAs with key roles in tumorigenesis such as miR-216b, which targets K-RAS oncogene in nasopharyngeal carcinoma and colorectal cells, were identified^13^,^14^. MiR-376c and miR-369-3p, which target the Insulin-like Growth Factor 1 Receptor (IGF1R) in melanoma^15^, and c-MYC in osteosarcoma, respectively, were also identified. Additionally, we detected upregulated miRNAs targeting important tumor suppressor genes in diverse neoplasia, such as miR-638 (BRCA1), miR-130b (TP53), miR-130b (CSF1), miR-142-3p (IL1A), and miR-301a (BIM, PTEN) (Table 2). However, the role of most of these miRNAs in breast cancer remains to be elucidated. Here, we focused in the study of miR-204, as its function has not been completely addressed in breast cancer. Previous reports indicate indicated that miR-204 is downregulated in diverse malignancies^16^,^17^,^18^,^19^,^20^,^21^,^22^,^23^,^24^,^25^,^26^ where it is associated with a poor prognostic and a more aggressive phenotype. A potential role for miR-204 in neovascularization processes no related to cancer has been reported. For instance, miR-204 modulates vascular remodeling in human pulmonary hypertension^27^,^28^, and loss of miR-204 is associated to corneal neovascularization in mice^29^.

Several miRNAs have been implicated in angiogenesis in diverse types of cancers^30^,^31^. Nonetheless; the potential biological role of most miRNAs in the angiogenesis regulation of breast cancer is poorly understood. To further define the functions of miR-204 in breast cancer we restored its expression by RNA mimics in MDA-MB-231 and MCF-7 breast cancer cells and analyzed its effects by diverse cellular approaches. The expression of miR-204 resulted in a reduction in cell proliferation, migration and invasion indicating that it functions as a tumor suppressor. Transcriptome analyses showed that a number of genes involved in cell proliferation, migration and angiogenesis, including CREB5, ARHGAP5, FOXC1 MAPRE2, RAB22A and SMAD4, were suppressed by miR-204. Particularly, we found the downregulation of the ANGPT1 and TGβR2 genes which are involved in cell proliferation, migration and angiogenesis in cancer cells^11^,^12^. Congruently, our data indicated that miR-204 was able to suppress angiogenesis *in vitro* through the direct targeting of ANGPT1 and TGβR2, indicating an important role in the neovascularization process. Recent studies showed that both ANGPT1 and TGFβR2 could be regulated by microRNAs^32^, however no previous involvement of miR-204 in angiogenesis in breast cancer has been described. ANGPT1 is a secreted glycoprotein, which binds to Tie2/Tie1 receptors expressed in vascular endothelium, and exerts downstream cellular effects required for the organization and maturation of newly formed vessels^33^ ANGPT1 also inhibits apoptosis, stimulates migration, and cell proliferation^34^,^35^,^36^. On the other hand, TGFβR2 is the ligand-binding receptor for all members of TGFβ family, and previous studies in mouse models have reported that loss of TGFβR2 expression in mammary fibroblasts is linked to tumor initiation and metastasis and to cell proliferation, and angiogenesis^37^. TGFβ pathway plays intricate roles in tumorigenesis behaving as a tumor suppressor at early stages of carcinogenesis as well as a tumor promoter at late stages^38^,^39^. The TGFβ pathway promotes tumor progression by inducing tumor growth, epithelial mesenchymal transition, invasion, and metastasis and plays important roles in physiological and pathological angiogenesis^40^. Notably, the TGFβ pathway regulates a number of miRNAs such as miR-29a which promotes angiogenesis in endothelium^41^. Taken altogether our results suggested that miR-204 plays a role in angiogenesis through the negative regulation of ANGPT1 and TGFβR2. Congruently, we found an inverse correlation between the expression of mR-204 and ANGPT1 and TGFβR2 in breast tumors and cancer cell lines. Intriguingly, in some cases, the expression levels of ANGPT1 and TGFBR2 did not correlated with miR-204 levels as is common when compare tissues and cell lines because the heterogeneity of cancer cells. The genetic background of breast cancer cell lines confers differential changes in the expression impacting biological processes resulting from mutational, transcriptional and epigenetic changes. We observed similar findings, particularly for breast cancer cell lines that express different levels of miR-204 and ANGPT21/TGBR2 which sometimes did not correlate because the heterogeneous genetic background of the cells. It is important to note that target gene levels could be finely coregulated by others microRNAs or additional genetic or epigenetic mechanisms which may differ between the breast cancer cells lines studied here which explains, in part, the discordance between miR-204 and ANGPT21/TGBR2 levels observed in a minority of cell lines analyzed here. We found higher ANGPT1 expression in MDA-MB-231, BT20, MCF-7, and ZR-75-1 cell lines, but not inT47D and SKBR3 cells, in comparison to non-tumorigenic MCF-10A and normal adjacent tissues (Fig. 7H). In a similar way high expression levels of TGFβR2 were observed in five breast cancer cell lines, except for ZR-75-1 cells, relative to MCF-10A and normal tissues. These observations are strengthened by the results of the integration-based approach for ANGPT1 and TGFβR2 expression in a larger cohort of breast cancer patients (n = 522) using datasets from TCGA which indicate that both proteins were downregulated in the half of samples (Fig. 7G). It will be expected that the targets levels should be inversely proportional to miR-204 expression, however we don’t found this behavior in less of half of cell lines analyzed, this could be due also to different regulation mechanisms of TGFβR2 and ANGPT1 expression level, which did not involve miRNAs. The mechanism behind the discordant expression between miR-204 and these two targets in specific cell lines remains to be defined.

Finally, here we provide evidences based on the functional analysis, including knockdown and recue assays, which highlight the role of miR-204/ANGPT1/TGFβR2 axis in angiogenesis. TGFβR2 gene silencing, but not ANGPT1, was able to impair cell proliferation and migration, whereas inhibition of both genes alters angiogenesis. In addition, rescue of ANGPT1 and TGFβR2 expression in MDA-MD-231 cells expressing miR-204 resulted in a partially restoration of angiogenesis *in vitro*. Thus it is reliable to propose that miR-204 may exert an anti-oncogenic activity in breast cancer cells by two pivotal mechanisms depicted in the working model: i) suppression of TGFβ pathway leading to cell proliferation, migration and angiogenesis repression, and ii) repression of ANGPT1 resulting in angiogenesis blockage (Fig. 9K). Although speculative and considering the role of miR-204 in angiogenesis, we propose that the implementation of microRNA mimics approaches may represent a potential tool for RNA-based breast cancer therapy.

## Methods

### Cell lines

Human breast carcinoma MDA-MB-231, MCF-7, MDA-MB-453, ZR-75 and T457-D cell lines were obtained from the American Type Culture Collection, and routinely grown in Dulbecco’s modification of Eagle’s minimal medium (DMEM) supplemented with 10% fetal bovine serum and penicillin-streptomycin (50 unit/ml; Invitrogen, Carlsbad, CA, USA).

### Animals

Female athymic nu/nu mice at 6–8 weeks of age were used in the experiments. All animals were maintained on 12 h light/12 h dark. Food and water were available *ad libitum* before to angioreactors implantation Mice were anesthetized with 100 mg/ml Ketamine HCL and 20 mg/ml Xylazine.

### Tissues collection

None of the patients recruited in this study received any antineoplastic therapy prior to surgery. After tumor resection clinical specimens were embedded in Tissue-Tek and snap frozen in liquid nitrogen at −80 °C. Pathologist confirmed the existence of at least 80% tumor cells in clinical specimens. All experimental protocols with human tissues were approved by the ethics committee of Institute of Breast Diseases-FUCAM.

### Ethics statements

The Institute of Breast Diseases-FUCAM, Mexico provided the locally invasive breast tumors and normal tissues collection. We confirm that all experiments were performed in accordance with relevant guidelines and regulations of FUCAM ethics committee. A signed informed consent was obtained from each participant or her representative prior to release for research use. For animal studies, all experimental protocols were carried out in accordance with the approved guidelines at CINVESTAV-IPN.

### RNA isolation

Tissues were lysed using a Tissue Ruptor (Qiagen Inc., Valencia, CA), and RNA was extracted using 1 ml Trizol (Invitrogen, Carlsbad, CA) per 50–100 mg tissue as described by the manufacturer. RNA integrity was assessed using capillary electrophoresis system Agilent 2100 Bioanalyzer. Samples with a RNA integrity number >6 were processed.

### MicroRNAs profiling

Expression analysis of 667 miRNAs in nine ductal breast tumors and normal adjacent tissues was performed by reverse transcription and quantitative real-time polymerase chain reaction (RT-qPCR) using the Megaplex TaqMan Low-Density Arrays (TLDAs) v2.0 system (Applied Biosystems. Foster City, CA) as described by the manufacturer. Briefly, 70 ng total RNA were retro-transcribed using stem-loop primers. In order to detect low abundant miRNAs, a pre-amplification step was included. The pre-amplified product was loaded into the TLDA and amplification signal detection was performed in the 7900 FAST real time thermal cycler (ABI). Tests were normalized using RNU48 and RNU44 as controls.

### Validation of microRNAs profiling data

The Cancer Genome Atlas (TCGA) was used to obtain the datasets of miRNA expression from 776 matched tumors and adjacent normal tissues available from TCGA data matrix (http://tcga-data.nci.nih.gov/tcga/dataAccessMatrix.htm). This dataset was compared with the miRNAs expression profile obtained here in the discovery cohort using TLDAs.

### Statistical analysis of microRNAs expression

MiRNAs levels were measured by RT-qPCR in TLDAs using the comparative Ct (2−ΔΔCt) method. All analyses were done using R (HTqPCR, gplots-bioconductor). The Ct raw data was determined using an automatic baseline and a threshold of 0.2. A fold change (log2 RQ) value >1.0 was used to define the differentially expressed miRNAs. An adjusted t-test was used to evaluate the significant differences in Ct values between tumoral and non-tumoral tissues. To identify sub-groups defined by miRNAs expression profiles, an unsupervised clustering analysis using Spearman correlation and average linkage was used.

### Reverse transcription and real-time polymerase chain reaction

Quantitative RT-PCR analysis of individual microRNAs was performed using MiRNA Assays (Applied Biosystems, Foster City, CA). 100 ng total RNA were reverse transcribed using a looped-RT specific primer, 0.15 μl dNTPs (100 mM), 1.0 μl reverse transcriptase MultiScribe (50 U/μl), 1.5 μl 10X buffer, 0.19 μl RNase inhibitor (20 U/μl) and 4.16 μl RNase-free water. Then, diluted retrotranscription reaction (1:15) was mixed with 10 μl master mix TaqMan (Universal PCR Master Mix, No AmpErase UNG, 2X), 7.67 μl RNase free water, and 1.0 μl PCR probe. PCR reaction was performed in a GeneAmp System 9700 (Applied Biosystems) as follows: 95 °C for 10 min, and 40 cycles at 95 °C for 15 s and 60 °C for 1 min. Tests were normalized using RNU44 and RNU48 as control.

### Prediction of miRNAs targets

MiRNAs target genes were predicted using TargetScan and PicTar software. Only gene targets predicted by the two algorithms were included in further analysis.

### MicroRNA-204 transfection

MiRNA-204 precursor (pre-miR-204, Life Technologies) and pre-miR-negative control scramble (AM17110, Life Technologies) were transfected at 30 nM in MDA-MB-231 and MCF-7 cells using siPORT amine transfection agent (Ambion). Briefly, pre-miR-204 was individually added to wells containing 1 × 10^7 ^cells cultured in DMEM for 48 h. Expression of miR-204 was evaluated by RT-qPCR at 48 h postransfection.

### Clonogenic assays

MCF-7 and MDA-MB-231 cells were transfected with pre-miR-204 (30 nM) and scramble (30 nM). Forty-eight hours after transfection, cells were trypsinized, manually counted and seeded in six-well plates (1000 cells per well) to form colonies in 1–3 weeks. A colony was defined to consist of at least 50 cells. After 1–3 weeks, the colonies were counted and experimental data were analyzed. At least three independent experiments were performed for each cell line and data were expressed as mean ±S.D. p < 0.05 was considered as statistically significant.

### Cell migration and invasion assays

Briefly, cells treated with the pre-miR-204 (30 nM), or scramble sequence (30 nM) were seeded in triplicate in a six-well plate and grown to 80% confluence. Twenty-four hours postransfection, a vertical wound was traced in the cell monolayer. After 12 and 24 h, cells were fixed with 4% paraformaldehyde and the scratched area was quantified. In transwell assays, chambers (Corning) with 6.5-mm diameter and 8-μm pore size polycarbonate membrane were used. MDA-MB-231 and MCF-7 cells (1 × 10^5^) transfected with pre-miR-204, or scramble were transferred to 0.5 ml serum-free medium and placed in the upper chamber, whereas the lower chamber was loaded with 0.8 ml medium containing 10% fetal bovine serum. The total number of cells that migrated into the lower chamber was counted after 24 h incubation at 37 °C. Cell invasiveness was evaluated using transwell chambers coated with a layer of extracellular matrix (BD Biosciences). MDA-MB-231 cells were treated with pre-miR-204 (30 nM) and 24 h postransfection, the invasive cells were fixed with 100% methanol, stained with 1% toluidine blue (Sigma) and quantified by manual counting in randomly selected areas. Experiments were performed three times by triplicate and results were expressed as mean  ± S.D. *p* < 0.05 was considered as statistically significant.

### Angiogenesis assays *in vitro*

Tubules formation assays based on the ability of human umbilical vein endothelial cells (HUVEC) to form three-dimensional capillary-like tubular structures *in vitro* were performed using the Gibco Angiogenesis Starter Kit (A1460901), which contains media and reagents optimized for culturing HUVEC on Geltrex LDEV-Free Reduced Growth Factor Basement Membrane Matrix to model the formation of endothelial cell tube networks. Procedures were as follows: Wells of a 24-well plate were coated with 50 μL of geltrex matrix and incubated at 37 °C for 30 min. Then, MDA-MB-231 cells (1 × 10^4^cells/well) transfected with pre-miR-204 (30 nM) or scramble (30 nM) negative control were added and cultured in complete DMEM medium. After confluence, medium was removed and HUVEC (1 × 10^4 ^cells) were co-cultured with MDA-MB-231 cells in DMEM without complement. Cultures of HUVEC alone or treated with VEGFA were used as negative and positive controls, respectively. After 24 h of co-culture, the formed tubules were observed under an inverted microscope (Iroscope SI-PH) and imaged. Branch points and tubular structures were individually counted by two observers. MDA-MB-231 cells (1 × 10^4 ^cells/well) silenced in ANGPT1 or TGFβR2 genes by RNA interference were also evaluated. Experiments were performed three times by triplicate and results were expressed as mean ± S.D. *p* < 0.05 was considered as statistically significant.

### Directed *in vivo* angiogenesis assay

Angiogenesis *in vivo* was evaluated using the DIVAA kit (Trevigen) with some modifications. Briefly, angioreactors were filed with 50,000 pre-miR-204 transfected or scramble control MDA-MB-231 cells embedded in 20 μl of basement membrane extract (BME). Angioreactors were incubated at 37 °C for 1 h. For positive controls, angioreactors were filled with BME supplemented with VEGF (18 ng/ml) plus FGF1 (60 ng/ml). Two angioreactors were implanted in each immunocompromised nude mice subcutaneously in the dorsal region, (2 mice for pre-miR-204, 2 mice for scramble and 2 mice for VEGF/FGF1). The angioreactors were removed after 9 days after implantation, angioreactors were photographed. Presence of blood vessels quantified using FITC-Lectin detection, fluorescence was determined in a plate reader as mean relative fluorescence units.

### DNA microarrays analysis

Global gene expression analysis was done for MDA-MB-231 cells transfected with pre-miR-204 (30 nM) or scramble (30 nM) using the NimbleGen array (Roche). RNA samples were used to synthesize double-stranded labeled cDNA using SuperScript Double-Stranded cDNA Synthesis Kit (Invitrogen) and NimbleGen One-Color DNA Labeling Kit. Samples were hybridized in NimbleGen array 12 × 135 K (12 × 135,000 features). After hybridization and washing, the processed slides were scanned using a NimbleGen MS200 Microarray Scanner. Raw data were extracted as pair files by NimbleScan software (version 2.5), background corrected and data were normalized. The probe level files and gene summary files were produced and imported into ANAIS software (Analysis of NimbleGen Arrays Interface) for further analysis. DNA microarrays data for 10 selected genes was validated by RT-qPCR using specific oligonucleotides.

### Gene Ontology (GO) analysis

In order to determine possible pathways affected by the modulated genes by miR-204 an enrichment an analysis was performed using Gene Ontology (GO) program DAVID 6.7. Gene Ontology (GO) is an international standardized classification system for gene function, which supplies a set of controlled vocabulary to comprehensively describe the property of genes and gene products. We analyzed the 3 classifications from GO: cellular component, molecular function and biological process using the default parameters.

### Luciferase reporter gene assays

The 3′UTR of ANGPT1 and TGFβR2 genes was amplified using specific primers and cloned downstream of luciferase gene into p-miR-report vector (Ambion). All constructs were verified through automatic sequencing. The recombinant pmiR-LUC-ANGPT1 and pmiR-LUC-TGFβR2 plasmids were transfected into MDA-MB-231 cells using lipofectamine 2000 (Invitrogen). At 3 h postransfection, the medium was replaced with complete fresh medium. 24 h after transfection, pre-miR-204 (30 nM) or pre-miR-negative control (scramble) were co-transfected with lipofectamine RNAi max (Invitrogen). Then 24 h after transfection, firefly and renilla luciferase activities were both measured by the Dual-Glo luciferase Assay (Promega) using a Fluoroskan Ascent™ Microplate Fluorometer (Thermo Scientific). Firefly luciferase activities were normalized with *Renilla reniformis* luciferase activities. *P* values for differences between control and pre-miR-204 transfected cells were determined by two-tailed Student’s t test.

### Western blots

Proteins were separated on 10% polyacrylamide gels and transferred to 0.2 μm nitrocellulose membrane (Bio-Rad) in transfer buffer (25 mM Tris, 192 mM glycine and 10% methanol). Membranes were dried and blocked for 60 min at room temperature with TBST-1X (137 mM NaCl, 20 mM Tris, 0.1% Tween-20, at pH 7.6) containing 5% BSA (Sigma-Aldrich) and incubated overnight at 4 °C with rabbit anti-GAPDH (1:2000 Cell Signaling), rabbit anti-ANGPT1 (1:2000 Abcam), rabbit anti-CREB5 (1:1000 Abcam), and rabbit anti-TGFβR2 (1:1000 Abcam) antibodies. Membranes were washed three times in TBST-1X and incubated with horseradish peroxidase-conjugated goat anti-rabbit IgG (1:2,500, Zymed). Signal was detected and developed using ECL Western blot detection reagent (Amersham).

### RNA interference assays

Four oligonucleotides (21–23 nt) encoding short hairpin RNAs (shRNAs) targeting the *ANGPT1* and *TGFβR2* genes were designed. To minimize the possibility of shRNAs off targeting effects, a nucleotide BLAST search was performed. The oligonucleotides codifying for specific shRNAs were cloned into pSilencer 5.1 U6 retro plasmid (Ambion) and sequences were confirmed by automatic sequencing. The resulting recombinant plasmids were dubbed as sh-ANGPT1 1.1, sh-ANGPT1 1.2, sh-TGFβR2 1.1 and sh-TGFβR2 1.2. The effectiveness of the constructs in gene silencing was tested by transient transfection of MDA-MB-231 cells (2 × 10^5^) using lipofectamine 2000 reagent (Invitrogen, 12566014) followed by ANGPT1 and TGFβR2 detection using Western blotting at 48 h post-transfection.

### ANGPT1 and TGFβR2 expression and rescue assays

The ANGPT1 (NM_001146.4) open reading frame (ORF) was amplified from human cDNA of MDA-MB-231 cells and by polymerase chain reaction (PCR) using the primers: 5′-CCCAAGCTTATGACAGTTTTCCTTTCCTTTGCT-3′ (forward) and 5′-CCGCTCGAGTGTGAACTCAAACGGCTCCA-3′ (reverse). The TGFβR2 (NM_001024847.2) ORF was amplified from human cDNA of MDA-MB-231 cells by PCR using the primers: 5′-CCCAAGCTT ATGGGTCGGGGGCTGCTCAGGG-3′ (forward) and 5′-CCGCTCGAGTTTGGTAGTGTTTAGGGAGCCG TCT-3′ (reverse). The ANGPT1 and TGFβR2 genes were orientally cloned into *Hind*III and *Xho*I restriction sites of pCDNA3 mammalian expression vector (Thermo Fisher Scientific) to generate pCDNA3-ANGPT1 and pCDNA3-TGFβR2 plasmids. All constructs were verified through automatic sequencing. 5 μg each of constructions were individually transfected into MDA-MB-231 cells using Lipofectamine 2000 (Invitrogen). After transfection Western blotting were performed to determine the overexpression of ANGPT1 and TGFβR2 proteins. For rescue assays, MDA-MB-231 cells (250,000) were transfected with miR-204 precursor or pre-miR-negative control (scramble). 24 h after miRNA transfection MDA-MB-231 cells were individually cotransfected with pCDNA3-ANGPT1 or pCDNA3-TGFβR2 constructions and after 48 h transfected cells (10,000) were prepared for angiogenesis assays as describe before. Briefly, cells were seed in BME coated well in 96 well plate in medium without supplement, and 24 h after HUVEC cells (10,000) were co-cultured with MDA-MB-231 cells as described above. Tube structures formation were analyzed and quantified 24 h after the co-cultures.

### Statistical analysis

Experiments were performed three times by triplicate and results were represented as mean ±S.D. One-way analysis of variance (ANOVA) followed by Tukey’s test were used to compare the differences between means. A *p* < 0.05 was considered as statistically significant.

## Additional Information

**How to cite this article**: Flores-Pérez, A. *et al*. Dual targeting of ANGPT1 and TGFBR2 genes by miR-204 controls angiogenesis in breast cancer. *Sci. Rep.* **6**, 34504; doi: 10.1038/srep34504 (2016).

## Supplementary Material

Supplementary Information

Supplementary Information

## Figures and Tables

**Table 1:** Clinical and pathological features of breast tumors analyzed by stem-loop reverse transcription-quantitative PCR in TaqMan low-density arrays.

Patient	Age (years)	Clinical stage	Tumor grade	Classification	Tumor size (mm)	Histological subtype	HER2	ER	PR
15	45	II	2	HER2	35	Infiltrating ductal carcinoma	+	−	+
30	41	I	2	Luminal B	20	*In situ* ductal carcinoma	+	+	+
75	50	IIB	2	HER2	25	Infiltrating ductal carcinoma	+	−	−
76	49	IIB	3	HER2	20	Infiltrating ductal carcinoma	+	−	−
78	81	IIIB	3	HER2	47	Infiltrating ductal carcinoma	+	−	−
85	47	IIA	3	HER2	27	Infiltrating ductal carcinoma	+	−	+
88	63	I	ND	Luminal A	15	Infiltrating ductal carcinoma	+	+	−
105	39	IIIC	2	HER2	23	Infiltrating ductal carcinoma	+	−	+
191	55	IIB	ND	HER2	39	Infiltrating ductal carcinoma	+	−	−

HER2, Human epidermal receptor 2. ER, Estrogen receptor. PR, Progesterone receptor. ND, Not determined.

**Table 2:** MicroRNAs modulated in locally invasive breast tumors.

	Fold change (log2 RQ)	RQ-value	p-value	*Locus*
Down-regulated miRNAs				
hsa-miR-204	−4.87	0.0342	0.003	9q21.12
hsa-miR-139-3p	−3.86	0.0691	0.005	11q13.4
hsa-miR-125b-2*	−3.54	0.0859	0.038	21q21.1
hsa-miR-486-5p	−3.53	0.0866	0.001	8p11.21
hsa-miR-541	−3.25	0.1054	0.032	14q32.31
hsa-miR-216b	−3.04	0.1213	0.018	2p16.1
hsa-miR-139-5p	−2.76	0.1481	0.001	11q13.4
hsa-miR-489	−2.60	0.1650	0.042	7q21.3
hsa-miR-1	−2.08	0.2373	0.021	20q13.33
hsa-miR-335	−2.04	0.2428	0.051	7q32.2
hsa-miR-376c	−1.93	0.2623	0.009	14q32.31
hsa-miR-132*	−1.89	0.2697	0.021	17p13.3
hsa-miR-132	−1.75	0.2973	0.010	17p13.3
hsa-miR-369-3p	−1.73	0.3009	0.006	14q32.31
hsa-miR-944	−1.72	0.3028	0.025	3q28
hsa-miR-488	−1.66	0.3157	0.051	1q25.2
hsa-miR-433	−1.62	0.3247	0.006	14q32.2
hsa-miR-424*	−1.60	0.3296	0.001	Xq26.3
hsa-miR-508-3p	−1.49	0.3566	0.030	Xq27.3
hsa-miR-218	−1.49	0.3568	0.003	4p15.31
hsa-miR-337-3p	−1.47	0.3612	0.044	14q32.2
hsa-miR-10b*	−1.45	0.3648	0.001	2q31.1
hsa-let7c	−1.42	0.3739	0.040	21q21.1
hsa-let7b	−1.34	0.3962	0.038	22q13.31
hsa-miR-376a	−1.32	0.4011	0.021	14q32.31
hsa-miR-26b*	−1.29	0.4093	0.006	2q35
hsa-miR-656	−1.26	0.4161	0.018	14q32.31
hsa-miR-95	−1.26	0.4165	0.038	4p16.1
hsa-miR-221*	−1.24	0.4243	0.016	Xp11.3
hsa-miR-543	−1.20	0.4361	0.014	14q32.31
hsa-miR-10b	−1.18	0.4420	0.015	2q31.1
hsa-miR-100	−1.18	0.4427	0.037	11q24.1
hsa-miR-432	−1.05	0.4816	0.035	14q32.2
hsa-miR-195	−1.03	0.4898	0.048	17p13.1
Up-regulated miRNAs				
has-miR-155	11.91	3849.69	0.019	21q21.3
hsa-miR-592	4.43	21.4937	0.003	7q31.33
hsa-miR-638	3.14	8.8009	0.035	19p13.2
hsa-miR-183*	3.08	8.4533	0.005	7q32.2
hsa-miR-21	2.73	6.6543	0.029	17q23.1
hsa-miR-130b	2.37	5.1594	0.044	22q11.21
hsa-miR-142-3p	2.26	4.7981	0.019	17q22
hsa-miR-708	2.22	4.6601	0.049	11q14.1
hsa-miR-181a*	2.16	4.4810	0.044	1q32.1
hsa-let7g*	2.10	4.2809	0.004	3p21.1
hsa-miR-148b*	2.10	4.2749	0.010	12q13.13
hsa-miR-18b	1.80	3.4850	0.035	Xq26.2
hsa-miR-454*	1.79	3.4608	0.007	17q23.2
hsa-miR-7	1.71	3.2647	0.031	9q21.32
hsa-miR-142-5p	1.68	3.2130	0.039	17q22
hsa-miR-431	1.60	3.0374	0.031	14q32.2
hsa-miR-301a	1.38	2.6071	0.024	17q22
hsa-miR-188-5p	1.32	2.5022	0.026	Xp11.23
hsa-miR-331-3p	1.19	2.2822	0.003	12q22
hsa-miR-425*	1.18	2.2718	0.041	3p21.31

**Table 3:** Clinical features of breast tumors analyzed for miR-204 expression by RT-qPCR.

Characteristics	Patients (n = 58)
**Classification**	
HER2 positive	5
Luminal A	25
Luminal B	7
Triple negative	17
Unknown	4
**Histology**	
*In situ* ductal carcinoma	17
Infiltrating ductal carcinoma	20
Infiltrating lobular carcinoma	8
Invasive mucinous carcinoma	3
Mixed carcinoma (ductal and lobular)	6
Others	4
**Clinical stage**	
I	8
II	27
III	19
IV	4
