# Supplementary material for: Dual targeting of ANGPT1 and TGFBR2 genes by miR-204 controls angiogenesis in breast cancer
Source: Sci Rep. 2016 Oct 5;6:34504. doi: 10.1038/srep34504 (PMC5050489; doi:10.1038/srep34504)
Supplement: Supplementary Information [file srep34504-s1.pdf]

**Supplementary information**

# Dual targeting of ANGPT1 and TGFBR2 genes by miR-204 controls angiogenesis in breast cancer

Ali Flores-Pérez, Laurence A. Marchat, Sergio Rodríguez-Cuevas, Verónica Bautista-Piña, Alfredo Hidalgo-Miranda, Elena Aréchaga Ocampo, Mónica Sierra Martínez, Carlos Palma-Flores, Miguel A. Fonseca-Sánchez, Horacio Astudillo-de la Vega, Erika Ruiz-García, Juan Antonio González-Barrios, Carlos Pérez-Plasencia, María L. Streber, César López-Camarillo

**Supplementary figure 1**. Validation of expression of 33 out 54 miRNAs detected by TLDAs (discovery cohort) using TCGA data sets (validation cohort) in 776 tumor and normal adjacent matched tissues. In bold are denoted 4 miRNAs with discordant expression between the discovery and validation cohort.

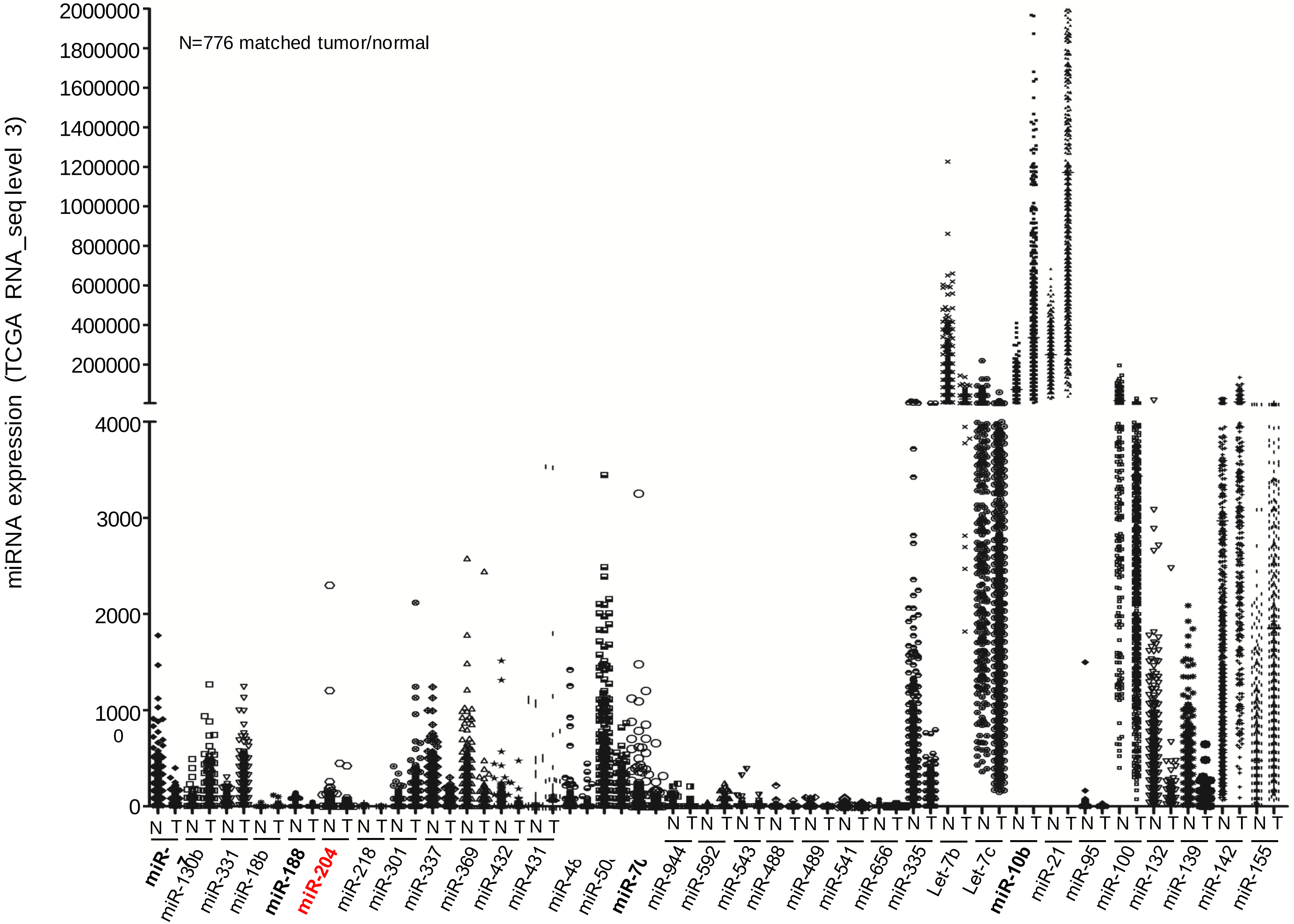

Supplementary figure 2. Restoration of miR-204 expression in MDA-MB-231 breast cancer cell line

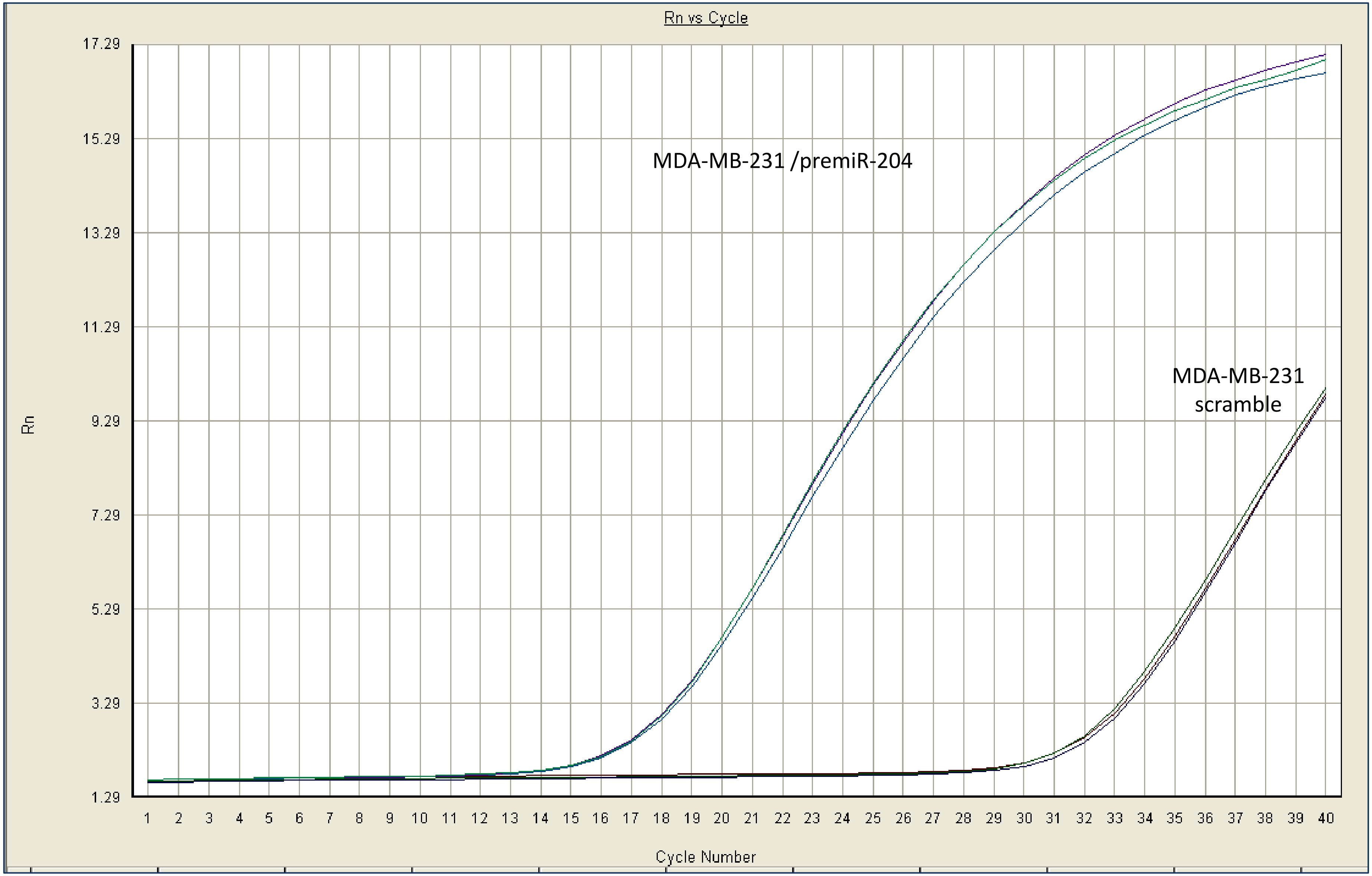

Supplementary figure 3. Validation of DNA microarrays analysis using RT-qPCR for ten selected genes.

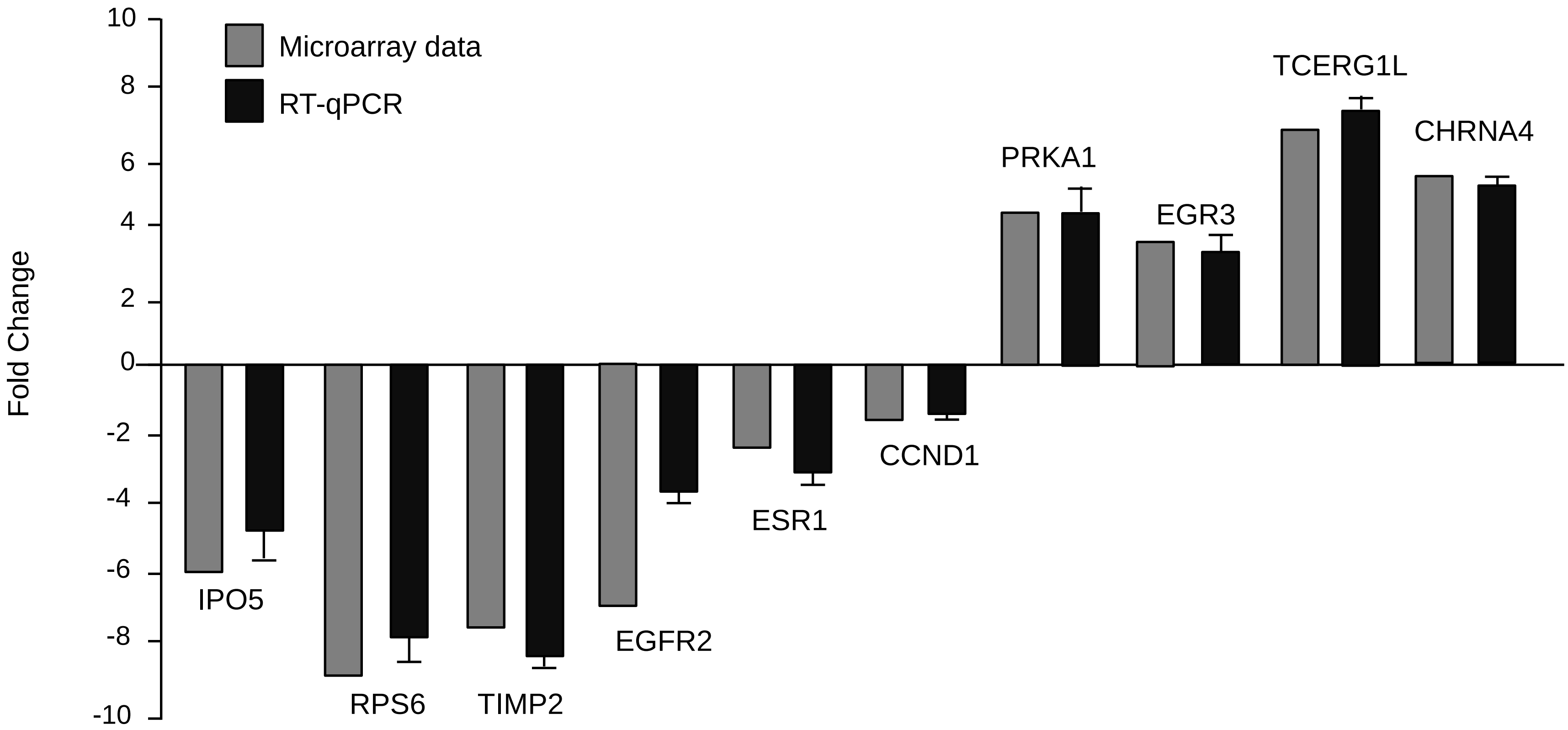

**Supplementary table 1**. MicroRNAs regulated in the complete set of breast tumors (9/9). Fold change (log2RQ) values are indicated for individual microRNAs in breast cancer patients (T).

| **Up-regulated** | **Number of samples found** | **Breast cancer patient (T)** | | | | | | | | |
|---|---|---|---|---|---|---|---|---|---|---|
| | | **T 15** | **T 30** | **T 75** | **T 76** | **T 78** | **T 85** | **T 88** | **T 105** | **T 191** |
| hsa-let-7g* | 9/9 | 1.23 | 11.38 | 5.39 | 5.08 | 5.65 | 3.79 | 10.40 | 4.15 | 5.70 |
| hsa-miR-148b* | 9/9 | 1.14 | 1.53 | 0.40 | 2.67 | 2.98 | 1.02 | 2.33 | 1.18 | 3.59 |
| hsa-miR-454* | 9/9 | 1.49 | 2.85 | 2.26 | 1.00 | 1.40 | 1.02 | 1.00 | 1.79 | 1.94 |
| hsa-miR-592 | 9/9 | 2.92 | 5.18 | 2.71 | 5.26 | 5.36 | 2.54 | 5.00 | 3.28 | 3.79 |
| | | | | | | | | | | |
| **Downregulated** | **Number of samples found** | **Breast cancer patient (T)** | | | | | | | | |
| | | **T 15** | **T 30** | **T 75** | **T 76** | **T 78** | **T 85** | **T 88** | **T 105** | **T 191** |
| hsa-let-7c | 9/9 | -3.65 | -2.21 | -3.30 | -2.29 | -2.38 | -6.17 | -1.20 | -1.89 | -0.84 |
| hsa-miR-100 | 9/9 | -3.41 | -2.72 | -2.16 | -1.01 | -1.36 | -4.29 | -1.07 | -1.21 | -1.26 |
| hsa-miR-10b* | 9/9 | -1.75 | -1.01 | -1.65 | -0.64 | -0.55 | -3.61 | -0.81 | -3.03 | -1.86 |
| hsa-miR-10b | 9/9 | -2.83 | -1.65 | -2.53 | -1.24 | -1.05 | -4.84 | -1.35 | -0.80 | -1.58 |
| hsa-miR-132 | 9/9 | -2.80 | -1.21 | -1.48 | -4.10 | -5.32 | -3.96 | -2.13 | -0.22 | -1.86 |
| hsa-miR-139-3p | 9/9 | -6.48 | -2.43 | -3.25 | -8.90 | -8.83 | -6.63 | -9.38 | -3.33 | -11.29 |
| hsa-miR-1 | 9/9 | -2.44 | -0.79 | -1.79 | -4.90 | -3.20 | -5.76 | -3.23 | -1.35 | -1.66 |
| hsa-miR-195 | 9/9 | -3.19 | -1.00 | -3.00 | -1.46 | -1.47 | -4.37 | -0.97 | -1.48 | -0.88 |
| hsa-miR-204 | 9/9 | -6.73 | -4.67 | -2.87 | -6.84 | -7.51 | -9.04 | -5.85 | -6.47 | -6.42 |
| hsa-miR-218 | 9/9 | -3.08 | -2.08 | -2.19 | -1.63 | -1.64 | -4.86 | -1.21 | -0.77 | -2.34 |
| hsa-miR-26b* | 9/9 | -2.13 | -0.40 | -1.48 | -2.34 | -2.16 | -2.92 | -0.14 | -2.29 | -0.07 |
| hsa-miR-376a | 9/9 | -2.05 | -2.18 | -2.36 | -1.13 | -1.27 | -5.16 | -1.35 | -1.15 | -3.04 |
| hsa-miR-376c | 9/9 | -1.60 | -2.33 | -2.36 | -1.88 | -2.08 | -5.64 | -2.40 | -1.20 | -3.62 |
| hsa-miR-424* | 9/9 | -5.16 | -3.19 | -0.74 | -0.12 | -0.18 | -2.88 | -1.91 | -4.21 | -2.68 |
| hsa-miR-432 | 9/9 | -1.34 | -0.76 | -1.15 | -1.34 | -0.40 | -3.98 | 0.35 | -2.60 | -0.91 |
| hsa-miR-433 | 9/9 | -2.49 | -2.25 | -0.98 | -3.32 | -2.03 | -5.41 | -1.34 | -1.08 | -3.47 |
| hsa-miR-489 | 9/9 | -2.42 | -2.04 | -3.78 | -4.13 | -4.26 | -5.67 | -2.91 | -3.34 | -4.22 |
| hsa-miR-656 | 9/9 | -1.51 | -0.46 | -2.26 | -2.01 | -2.96 | -2.28 | 0.32 | -2.59 | -0.24 |
| hsa-miR-944 | 9/9 | -0.99 | -1.80 | -2.55 | -0.94 | -1.25 | -3.69 | -1.30 | -1.66 | -2.52 |

| Cellular pathway KEGG | Total genes | -ln (p-value) |
|---|---|---|
| MAPK signaling pathway | 114 | 22.63 |
| Focal adhesion | 90 | 19.83 |
| Wnt signaling pathway | 75 | 22.37 |
| Axon guidance | 71 | 28.93 |
| Ubiquitin mediated proteolysis | 63 | 15.39 |
| Colorectal cancer | 54 | 27.37 |
| Prostate cancer | 52 | 21.86 |
| Adherens junction | 48 | 27.51 |
| TGF-beta signaling pathway | 48 | 15.61 |
| ErbB signaling pathway | 47 | 15.38 |

**Supplementary table 2.** Cellular pathways potentially affected by microRNAs deregulated in breast tumors

| Gene | Sequence (5' - 3') | Product length (bp) | Nucleotide position |
|---|---|---|---|
| GAPDH | FW- CCCCACCACACTGAATCTCC<br>RV- GTACATGACAAGGTGCGGCT | 90 | 1266-1355 |
| IPO5 | FW- AGGAGAAATGCACGAGGCAA<br>RV- TTAAGGCCCTTCACGCAGAG | 173 | 3134-3306 |
| RPS6 | FW- TGTTACTCCACGTGTCCTGC<br>RV- AAGTCTGCGTCTCTTCGCAA | 166 | 579-744 |
| TIMP2 | FW- AGCTTTGCTTTATCCGGGCT<br>RV- ATGCTTAGCTGGCGTCACAT | 175 | 1868-2042 |
| EGFR2 | FW- GGTGGCATTTAGGGGTGACT<br>RV- CAAGGGAACAGGAAATATGTCG | 173 | 1365-1537 |
| ESR1 | FW- ACTCAACAGCGTGTCTCCG<br>RV- CACATTTTCCCTGGTTCCTGTAG | 173 | 529-701 |
| CCND1 | FW- GCTGTAGTGGGGTTCTAGGC<br>RV- GGCACGCTACGCTACTGTAA | 160 | 1626-1785 |
| PRKAA1 | FW- AGTGTCGCTTTTTGAATAGTTTG<br>RV- AGCAGTCTAGGCCAACAAGC | 243 | 661-903 |
| EGR3 | FW- GGTGACCATGAGCAGTTTGC<br>RV- TAGGTCACGGTCTTGTTGCC | 225 | 388-612 |
| ESR1 | FW- CGCTGCGTCGCCTCTAA<br>RV- TCCAGCTCGTTCCCTTGGAT | 182 | 124-305 |
| TCERG1L | FW- TACAGGTTTCCAAGGGTGGC<br>RV- TGCAAACTCCGGGCATACAA | 162 | 2405-2528 |
| CHRNA4 | FW- AATGTCACCTCCATCCGCATC<br>RV- GTCAGCATTGTTGTAGAGGG | 72 | 550-598 |
| ANGPT1 | FW- CGTGAATCTGGAGCCGTTTG<br>RV- AGTAGTTTGAAGCACAGCAAGC | 141 | 3'UTR 200-341 |
| TGFBR2 | FW- TGTCAGAGGATACTGTGGCTTG<br>RV- TGAGTACAGCTGAAGTGTTCCAT | 162 | 3' UTR 2250-2412 |
| CREB5 | TCTTCAAAGACTGGCTTTCATTTT<br>AAAGCACCAAAGCGTTTCCC | 125 | 3' UTR 150-275 |

**Supplementary table 4.** Primers used to validate DNA microarrays data.

**Supplementary table 5.** Cell proliferation and migration genes modulated by miR-204

| Expression | Biological Process | Number of genes | Gene Symbol |
|---|---|---|---|
| Downregulated | Proliferation | 38 | E2F1, EXO1, TXNIP, CLSPN, DBF4B, PDS5A, PDPN, TIPIN, SKP2, CDK4, TACC1, TACC2, CDT1, PSMA2, TUBB, EREG, NPM1, **TGFBR2*, SMAD4*** CHTF18, **MAPRE2*,** FANCA, DSCC1, CDK4, ID2, NPM1, FOXC1, ASNS, ID3, GPR3, **CREB5*,**EXTL3, AKT1S1, **ARHGAP5*,** SPTBN1, NEFL, CDH4,**RAB22A*** |
| | Migration/Invasion | 24 | CAP2, MARK1, LLGL1, HOOK3, TACC2, EPB41L2, CORO2B, TUBB, ANK3, NPM1, CAP1, NEFL, LCP1, TUBA8, KIF21A, TACC2,FMOD, EID2, ID1, **TGFBR2*, SMAD4*, FOXC1*, FRAS1*, LRP8*** |
| Upregulated | Proliferation | 32 | btg1, GJA1, BDKRB2, TRIB1, GPNMB, ptch1, Wisp2, Dbp, BTG4, Hyal1, Nppc, mmp7, il6r, irs2, Spn, KCTD11, ESR2, egln3, TNFRSF14, APC, nupr1, ADRA1A, RARRES1, Hyal1, LAMA5, Ang, Kiss1r, Ifitm1, Hyal1, Scgb3a1 ,LAMA5 ,GPNMB |
| | Migration/Invasion | 29 | TMEM8B, RET, fn1, GPNMB, ITGA1, RHOB, ITGA7, CNTN2, Cntn4, Wisp2, ROR2, Itgax, PCDHB9, PCDHB10, PCDHB11, CD72, PCDHB5, Wisp2, Nedd9, sspN, CDH1, fn1, APC, RHOB, Selplg, LAMA5, GPNMB, LAMA5, CCR1 |

*Genes with miR-204 binding sites

| Primer name | Sequence 5´-3´ | Nucleotide position in target gene |
|---|---|---|
| ANGPT1.1-S | GATCCGGATTTCAGAAGCCTAATTCCTTCAAGAGAGGAATTAGGCT TCTGAAATCCTTTTTT GGAAA | 2533-2554 |
| ANGPT1.1-AS | AGCTTTTCCAAAAAAGGATTTCAGAAGCCTAATTCCTCTCTTGAAG GAATTAGGCTTCTGAAATCCG | 2533-2554 |
| ANGPT1.2-S | GATCCGCCATAATGAACTGTAGTACATTCAAGAGA TGTACTACAGTTCATTATGGCTTTTTTGGAAA | 2617-2638 |
| ANGPT1.2-AS | AGCTTTTCCAAAAAAGCCATAATGAACTGTAGTACATCTCTTGAATG TACTACAGTTCATTATGGCG | 2617-2638 |
| TGFβR2 1.1-S | GATCC GGAGGAGAAGATTCCTGAAGA TTCAAGAGA TCTTCAGGAATCTTCTCCTCC TTTT TT GGAAA | 2041-2062 |
| TGFβR2 1.1-AS | AGCTTTTCCAAAAAAGGAGGAGAAGATTCCTGAAGATCTCTTGAAT CTTCAGGAATCTTCTCCTCCG | 2041-2062 |
| TGFβR2 1.2-S | GATCCGGTGTTAAGATTTGAAGTTGGTTCAAGAGACCAACTTCAAA TCTTAACACCTTTTTTGGAAA | 3566-3587 |
| TGFβR2 1.2-AS | AGCTTTTCCAAAAAAGGTGTTAAGATTTGAAGTTGGTCTCTTGAAC CAACTTCAAATCTTAACACCG | 3566-3587 |

**Supplementary table 6.** Primers used to silencing ANGPT1 and TGFβR2 gene expression
